# Developing an Educational Program for Ultrasound Hip Screening during Newborn and Infant Home Visits: A Protocol Paper

**DOI:** 10.3390/nursrep14010012

**Published:** 2024-01-08

**Authors:** Kyoko Yoshioka-Maeda, Chikako Honda, Hiroshige Matsumoto, Takeshi Kinjo, Kenta Fujiwara, Kiyoshi Aoki

**Affiliations:** 1 Department of Community Health Nursing, Division of Health Sciences and Nursing, Faculty of Medicine, The University of Tokyo, Bunkyo-ku, Tokyo 113-0033, Japan; hchika-tky@g.ecc.u-tokyo.ac.jp (C.H.); hiroshige-tky@g.ecc.u-tokyo.ac.jp (H.M.); 2 Department of Orthopedic Surgery, Okinawa Prefectural Nanbu Medical Center and Children’s Medical Center, Haebaru Town, Okinawa 901-1193, Japan; tk95029@yahoo.co.jp; 3 Doi Orthopedic Clinic, Takatsuki City 569-0803, Japan; kenta.fujiwara@ompu.ac.jp; 4 Department of Orthopedic Surgery, Asahigawasou Rehabilitation and Medical Center, Okayama 703-8207, Japan; gairai@asahigawasou.or.jp

**Keywords:** community, educational program, hip screening, nurse, physical assessment, ultrasonography

## Abstract

Ultrasound hip screening is suitable for the early identification of developmental dysplasia of the hip (DDH). Newborn and infant home visits are good opportunities for hip screening in the community, but studies focusing on nurse-led screenings are lacking. Based on a pre–post design, this study aims to develop and evaluate an ultrasound training program to improve nurses’ assessment skills in detecting DDH cases during newborn and infant home visits. Said educational program will include e-learning, hands-on seminars, and clinical training. The primary outcome will be the success rate of imaging standard planes (standardized images for hip assessment) in clinical training. The secondary outcomes will include knowledge test results, objective structured clinical examination scores, time required for imaging, and inter-rater reliability between nurses and physicians. The educational program will address the issue of missed and late detection of DDH cases in resource-limited communities. This study will demonstrate the feasibility of procedures and the effectiveness of the educational program in 2024. The protocol was registered in the University Hospital Medical Information Network Clinical Trial Registry before starting the study (no. UMIN000051929, 16 August 2023).

## 1. Introduction

With more attention being paid globally to the prevention of child maltreatment, interest in the issue of hip dislocation in children is diminishing [1]. Developmental dysplasia of the hip (DDH) is associated with several risk factors, including being a woman, breech presentation, positive family history, and winter births [2,3]. According to the latest literature review published in 2023, the prevalence of DDH is approximately 1.4%, with a wide range of ethnic and cultural differences [4]. Its early detection maximizes the treatment effect by reducing medical expenditure and preventing children’s gait abnormalities [5,6]. Traditionally, physical examinations, including Ortolani and Barlow tests, are used to detect DDH cases with click sign, leg length discrepancy, and limited abduction without which experts cannot perfectly identify such cases [7]. Plain radiography is also used to detect hip dislocation, with ultrasonography being an appropriate measure for the early detection of DDH without radiation exposure [8,9,10]. Although pediatric orthopedic surgeons have developed hip screening systems in hospitals or clinics, community-based hip screening using ultrasound still needs to be further explored. 

Under the controversial debate over whether universal or selective hip screening is better [11,12], universal screening was strongly recommended in an international consensus paper [13]. In Japan, more than 90% of local governments conduct maternal and child health services [14], presenting a good opportunity for universal hip screening in communities. Newborn and infant home visits are a crucial healthcare strategy for supporting maternal and child health in each community [15]. Public health nurses and midwives have been conducting home visits for newborns and infants since the 1960s [16]. When the use of traditional Japanese-style diapers and baby clothing contributed to DDH as they extended infants’ legs, medical doctors and nurses educated caregivers on how to recommend a new diaper style that maintains the natural flex position of infants’ legs during home visits [17,18]. With the success of this health education program, the incidence of DDH in Japan has significantly decreased and now stands at approximately 0.1% [19]. However, despite home visits and infant health checkups being conducted, a nationwide survey revealed that approximately 15% of DDH cases were diagnosed after the child turned one year old [20]. The national government emphasized the necessity of finding DDH cases in each community [21]. 

The aforementioned consensus paper argues that ultrasound screening should be performed at less than six weeks of age [13], while others argue that it should not be performed before six weeks of age as the maternal estrogen-induced physiological laxity improves by the age of six weeks [22]. In Japan, screening opportunities around six weeks include one-month checkups at hospitals and clinics and newborn visits by nurses. While both opportunities for screening should be utilized, there are advantages to newborn visits in disseminating ultrasound screening. First, compared to one-month checkups, which are performed at private expense, newborn visits, which are conducted by the local government based on the legal program, would enable universal dissemination of the new technology. Second, newborn visits are generally performed between four and eight weeks, which could be a more appropriate timing for screening. Third, it is primarily pediatricians who conduct one-month checkups, not orthopedic surgeons who take initiative in ultrasound screening, making it difficult to disseminate the use of ultrasound screening. However, improving the accuracy of hip screenings in community settings is a big challenge because each local government educates nurses based on local autonomy and has limited medical resources. 

Early detection of DDH could improve the affected individuals’ quality of life and reduce their medical expenditures. Rather than manual assessments, nurses can identify suspicious DDH cases more accurately and quickly by ultrasound. Point-of-care ultrasound is an innovative and non-invasive tool for conducting physical assessment in clinical, resource-limited rural, and community settings [23,24,25]. Nurses can use it to assess abnormal cases of cardiovascular, respiratory, and gastrointestinal, and obstetrics and gynecology diseases, as well as soft tissue and joint fluid issues [23,26,27]. Using point-of-care ultrasound for the early detection of medical issues can save time, reduce radiation exposure and medical costs, and improve patients’ outcomes [28]. However, no studies have focused on conducting ultrasound hip screening by nurses in community settings [29]. Therefore, to enhance the early detection of DDH cases, this study aims to develop and evaluate an ultrasound training program to improve nurses’ assessment skills regarding DDH cases in newborns and infants during home visits. 

## 2. Materials and Methods

### 2.1. Study Design and Setting

**Figure 1:**
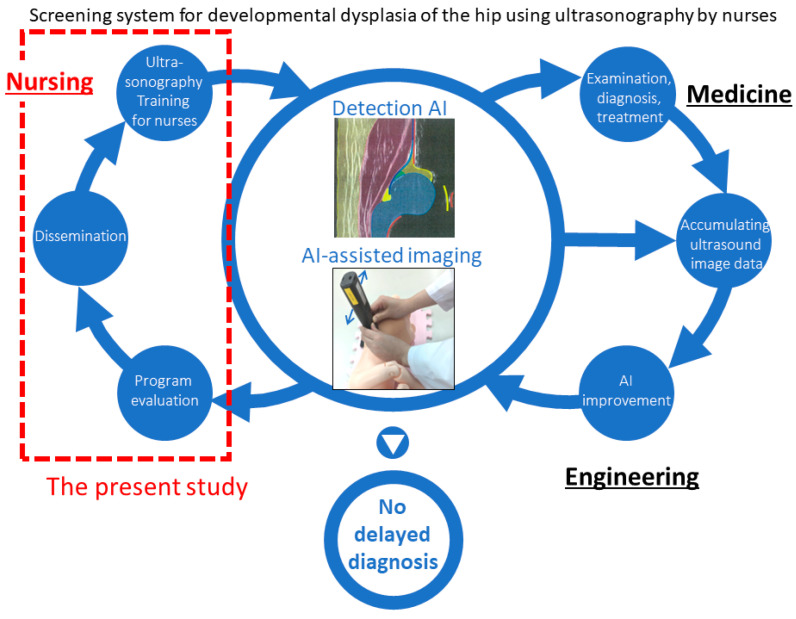
Schematic models of the community-based ultrasound hip screening system for preventing overlooked DDH cases in newborn and infant home visits [30]. Note: The area bounded by the dotted line is the current study. The reference number in the caption for the publication in the image was [33] (Aoki, K., & Kinjo, T., 2021).

This study is part of a research project that prevents cases of DDH from being overlooked in community settings for newborns and infants (Figure 1) [30]. Based on previous research [10,31,32] and a discussion with medical experts who supervised ultrasound hip screening at hands-on seminars conducted by the Japanese Society of Orthopedic Ultrasonics (T.K., K.F., K.A.), we developed an educational program for nurses with a pre-post design. 

This study will be started in Okinawa Prefecture, Japan. Okinawa is a rural area with several remote islands and limited medical resources.

The protocol was registered in the University Hospital Medical Information Network Clinical Trial Registry (UMIN-CTR) before starting the study (no. UMIN000051929, 16 August 2023), which the International Committee of Medical Journal Editors approved. 

### 2.2. Participants

Participants will be recruited using snowball sampling. We will recruit approximately 30–40 nurses to consider the feasibility and limited budget. The inclusion criteria are that participants must be public health nurses, midwives, or registered nurses (any license is acceptable) who have conducted newborn and infant home visits. The exclusion criteria are individuals who are health volunteers for infant home visits and do not have a nursing license. 

Participant demographic data will be collected at study registration, including age, sex, job title, department, license, years of public health experience, years working in maternal and child health, and years of clinical experience in a hospital setting.

### 2.3. Intervention

The research team in charge of developing the educational program consisted of researchers in public health nursing and pediatric orthopedic surgeons. Before developing the materials, the first and second authors participated in a hands-on seminar on DDH assessment using ultrasound on 25–26 February 2023. The Japan Society of Orthopedic Ultrasonics organized this seminar. Researchers (K.Y.M., C.H. and H.M.) also participated in the nursing ultrasound hands-on seminar focusing on kidney and colon assessment on 23 July 2023. The Research Institute for Next-Generation Nursing Education (RINGNE) was in charge of providing this seminar in Japan [34]. We developed the program contents and timeline based on the skills and knowledge gained from these hands-on seminars.

The theoretical framework is Gagne’s “analysis, design, development, implementation, and evaluation model” [35]. Based on the experience gained from attending the hands-on seminar and previous research [10,31,32,33], we developed the education and training program as follows: (1) e-learning; (2) hands-on seminars; and (3) clinical training (Table 1). 

The 90 min e-learning course comprises five modules: ultrasound basics, anatomy of the hip joint, principles of the Graf method and ultrasound image reading [10], practice of the Graf method [10], and ultrasound practice and health education in the newborn home visit. The ultrasound basics module incorporates an existing module from the RINGNE, which is commonly used in nursing education in Japan [34]. The materials including anatomy of the hip joint, principles of the Graf method [10], and ultrasound image regarding its practice were developed by the co-authors (K.A., K.F.) [33]. They are expert orthopedic pediatricians, renowned for their extensive experience in training medical doctors in ultrasound hip screening at the Japan Society of Orthopedic Ultrasonics. Researchers (K.Y.M., C.H. and H.M.), with experience in public health nursing and in conducting newborn and infant home visits, developed the materials for home visits and health education. The co-authors (K.A., K.F. and T.K.) validated all the materials and programs with the instructors’ perspectives at the Japan Society of Orthopedic Ultrasonics. Further, three nursing experts specializing in ultrasound assessment education at the RINGNE helped in developing these materials. 

Participants (nurses) will take the e-learning course after study registration, followed by an on-site hands-on seminar. The 105 min hands-on seminar comprises a lecture and practical training using phantoms. The phantom reproduces the normal hip joint. After training with the phantom, skills are evaluated by Objective Structured Clinical Examination (OSCE). Participants who pass the OSCE can proceed to the next step.

Finally, clinical training will be provided. The hip joints of infant volunteers within approximately four months, recruited by opportunity, will be examined. The first half of the clinical training will assess infants with physician supervision and guidance. In the second half of the training, participants will independently assess another infant without supervision. 

The ultrasound device *iViz air Ver.5 linear* (Fujifilm, Tokyo, Japan) will be used in all the training and examinations to standardize the procedures and readings. This device weighs 197 g, is easy to carry, and has been introduced to many home visiting nurses in Japan [31].

### 2.4. Instruments and Measures

#### 2.4.1. E-Learning

Knowledge tests on the content of each module will be incorporated for knowledge retention and evaluation. The test will evaluate the participant’s ability to distinguish normal/abnormal ultrasound images.

#### 2.4.2. Hands-On Seminar

The OSCE, which follows a practical training using a phantom, will evaluate skills in explaining ultrasound examinations, imaging, explaining assessment results to caregivers, and providing health education on DDH prevention in the newborn visit setting, by global rating (1–6) and checklist grading (0–100). 

#### 2.4.3. Clinical Training

Both medical-supervised and unsupervised examination training will be evaluated based on the success rate of imaging (0–100%), time required for imaging (seconds), and inter-rater reliability (0–1; agreement between the nurse’s assessment and the physician’s diagnosis). The assessment results from manual examination by a nurse, the results from ultrasound examination by a nurse, and the diagnostic results from a physician will be collected.

The primary outcome of this study will be the success rate in the unsupervised examination. 

### 2.5. Statistical Analysis

Knowledge test results, OSCE scores, success rates of standard plane imaging, and time required for imaging will be reported using descriptive statistics. Inter-rater reliability will be reported using the prevalence adjusted bias adjusted kappa for each participant. The sensitivity and specificity of manual examination nurse and ultrasound examination by a nurse will be compared, with the diagnostic results from a physician as the gold standard.

### 2.6. Ethical Considerations

This study will be conducted following the Declaration of Helsinki. Ethical approval was obtained from the primary investigator’s ethics review committee (Research Ethics Committee of the Faculty of Medicine of the University of Tokyo, no. 2023101NI). To protect participants’ privacy, the authors will explain the study aim and procedures. Only participants who provide informed consent, whether by themselves or their surrogate, can participate in this study. To protect participants’ privacy, we will use pseudonyms throughout the study.

## 3. Conclusions

The nursing educational program, which focuses on ultrasound hip screening, will help improve nurses’ assessment skills and knowledge of suspicious DDH cases in newborns and infants during home visits. The current findings will provide crucial evidence to support the nursing profession in conducting ultrasound hip screening and in promoting accurate assessments during community-based newborns and infants home visit. The educational program addresses the issue of missed and late detection of DDH cases in resource-limited communities. This study will demonstrate the feasibility of procedures and the effectiveness of the educational program in 2024. 

## Figures and Tables

**Table 1:** Table of contents of the education and training program.

	Contents	Time (Minutes)	Materials/Subjects	Evaluation Measurement
1	E-learning:			
	∙ Ultrasound basics	25	RINGNE	∙ Knowledge test for each module
	∙ Anatomy of the hip joint	15	Original (K.A., K.F.)	
	∙ Principles of the Graf method and ultrasound image reading	15	Original (K.A., K.F.)	
	∙ Practice of the Graf method	10	Original (K.A., K.F.)	
	∙ Ultrasound practice and health education in the newborn home visits	25	Original (K.Y.M., C.H., H.M.)	
2	Hands-on seminar			∙ OSCE (operation of ultrasound equipment, technical proficiency)
	∙ Lecture	45	Original (K.Y.M., C.H., H.M.)	
	∙ Practical training using phantom	60	Phantom (Kyoto Kagaku Co., Ltd., Kyoto, Japan)	
3	Clinical training			∙ Success rate of imaging ∙ Time required for imaging ∙ Assessment results from manual examination by a nurse; results from ultrasound examination by a nurse; and diagnostic results from a physician
	∙ Medical-supervised examination	30	Healthy volunteers within four months of age	
	∙ Unsupervised examination	90		

RINGNE: Research Institute for Next-Generation Nursing Education, OSCE: Objective Structured Clinical Examination. Time (minutes) for hands-on seminars and clinical training indicates training duration per participant in case of five participants per session.

## Data Availability

Not applicable.
